# MHD Stagnation-Point Flow and Heat Transfer with Effects of Viscous Dissipation, Joule Heating and Partial Velocity Slip

**DOI:** 10.1038/srep17848

**Published:** 2015-12-09

**Authors:** Mohd Hafizi Mat Yasin, Anuar Ishak, Ioan Pop

**Affiliations:** 1 School of Mathematical Sciences, Faculty of Science and Technology, Universiti Kebangsaan Malaysia, 43600 UKM Bangi, Selangor, Malaysia; 2 Department of Mathematics, Babeş-Bolyai University, 400084 Cluj-Napoca, Romania

## Abstract

The steady two-dimensional stagnation-point flow and heat transfer past a permeable stretching/shrinking sheet with effects of viscous dissipation, Joule heating and partial velocity slip in the presence of a magnetic field is investigated. The partial differential equations are reduced to nonlinear ordinary differential equations by using a similarity transformation, before being solved numerically by shooting technique. Results indicate that the skin friction coefficient and the local Nusselt number increase as magnetic parameter increases. It is found that for the stretching sheet the solution is unique while for the shrinking sheet there exist nonunique solutions (dual solutions) in certain range of parameters. The stability analysis shows that the upper branch solution is stable while the lower branch solution is unstable.

The flow and heat transfer of a viscous (Newtonian) and non-Newtonian fluid past a static flat plate is a classical problem and has been studied by many authors (see Schlichting and Gersten^1^, White^2^, Pop and Ingham^3^ and Bejan^4^). It seems that Crane^5^ was the first who has studied the viscous and incompressible fluid over a stretching sheet obtaining a closed form analytical solution. This paper has been extended by different authors taking into account different effect, such as, suction/injection, radiation, etc. Further, Miklavčič and Wang^6^ studied the viscous flow induced by a shrinking sheet. Existence and (non) uniqueness solutions were proven. Exact solutions, both numerical and in closed form were obtained. The industrial applications of stretching/shrinking sheets are numerous such as aerodynamic extrusion of plastic sheets, the boundary layer along a liquid film, condensation process of metallic plate in cooling bath and glass, and also polymer industries, etc. It is worth mentioning here that the shrinking sheet flow is essentially a backward flow and it shows physical phenomena quite distinct from the stretching sheet flow (Goldstein^7^).

Flow and heat transfer over a stretching/shrinking sheet near a stagnation point has attracted the interest of many researchers. Ishak *et al.*^8^ investigated the stagnation-point flow and heat transfer over a shrinking sheet in a micropolar fluid. Bachok and Ishak^9^ studied numerically the stagnation-point flow towards an exponentially stretching/shrinking sheet immersed in a viscous fluid. Then, an unsteady two-dimensional flow and heat trasnfer of a viscous fluid near a stagnation-point over a shrinking sheet in the presence of time-dependent free stream have been analysed by Mahapatra and Nandy^10^. Suali *et al.*^11^ studied the similar problem but with prescribed surface heat flux. Next, Chen^12^ presented the unsteady mixed convection flow over a stretching sheet in the presence of velocity and thermal slips near the stagnation-point. The steady stagnation-point flow in the presence of chemical reaction past a stretching/shrinking cylinder was done by Najib *et al.*^13^. Zaimi *et al.*^14^ investigated the flow and heat transfer over a stretching/shrinking sheet in a nanofluid and reported the existence of dual solutions for a certain range of parameter. Das^15^ considered a steady two dimensional laminar boundary layer stagnation point flow in a micropolar fluid towards a shrinking sheet in the presence of magnetic field. Nandy^16^ and Lok *et al.*^17^, respectively, investigated the magnetohydrodynamic (MHD) stagnation point flow past a stretching and shrinking sheets.

The aim of this study is to investigate the effects of viscous dissipation, Joule heating and partial velocity slip of a viscous, incompressible and electrically conducting fluid near the stagnation point on a stretching/shrinking sheet in the presence of magnetic field near the stagnation point when suction and injection is taken into consideration. A stability analysis is performed to investigate the stability of the dual solutions and thus determine which solution is stable and physically realiable.

## Problem formulation

Consider a steady two dimensional, electrically conducting viscous and incompressible fluid over a permeable stretching/shrinking sheet coinciding with the plane 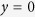, and the flow being confined to 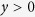. This problem also considers the effect of viscous dissipation, Joule heating and partial slip. The flow is generated by the stretching/shrinking effect along the 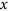
**-**axis. It is assumed that the velocity of the outer (inviscid) flow is 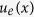, while that of the stretching/shrinking is 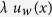 with *λ* being a positive constant for the stretching sheet and *λ* a negative constant for the shrinking sheet. The constant applied magnetic field, parallel to the 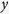 axis is 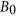. Also, the surface mass transfer velocity is 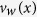 with 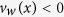 for suction and 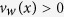 for injection. Further, it is assumed that the surface temperature is 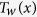, while the constant ambient temperature is 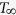. Under these conditions, the governing boundary layer equations are


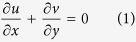



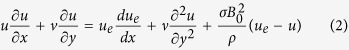



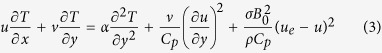


The boundary conditions of Eqs. (1)–(3), 14, 15 are


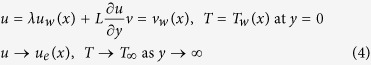


where 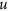 and 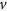 are the velocity components in the 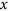 and 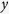 directions, respectively, 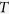 is the fluid temperature, 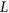 is the velocity slip length, 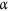 is the thermal conductivity, 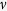 is the kinematic viscosity, 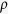 is the density, 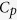 is the specific heat at constant pressure and 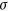 is the electrical conductivity.

In order that Eqs. (1) to (3) admit similarity solutions, we assume that 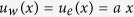 and 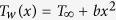, where 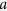 and *b* are positive constants. The momentum and energy equations (2) and (3) can then be transformed into the corresponding nonlinear ordinary differential equations by the following similarity transformation


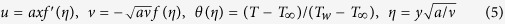


where prime denotes differentiation with respect to 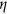. Based on Eq. (5), the mass transfer velocity 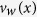 is given by


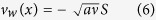


where 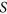 is the constant mass transfer velocity parameter with 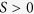 for suction and 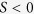 for injection.

Substituting Eq. (6) into Eqs. (2) and (3), we get the following system of nonlinear ordinary differential equations:


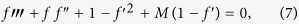



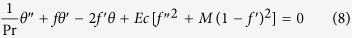


and the boundary conditions (4) become


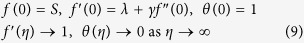


where 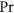 is the Prandtl number, 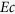 is the Eckert number, *M* is the constant magnetic parameter and 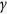 is the velocity slip parameter, which are defined as


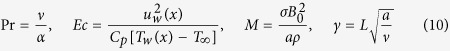


Physical quantities of interest are the skin friction coefficient 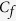 and the local Nusselt number 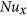, which are given by


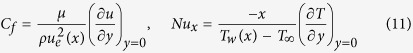


Using variables (5), we obtain


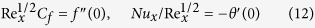


where 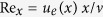 is the local Reynold number.

## Stability of solutions

We mention that there are several papers that have performed the stability analysis to determine which solution is stable and physically reliable, such as Merkin^18^, Weidman *et al.*^19^, Roşca and Pop^20^,^21^, Sharma *et al.*^22^ and Mansur *et al.*^23^. As in these papers, in order to perform a stability analysis, we consider the unsteady problem. Equation (1) holds, while (2) and (3) are replaced by


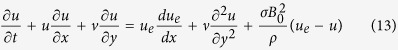



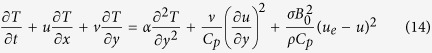


where *t* denotes the time. Based on the variables (5), we introduce the following new dimensionless variables:


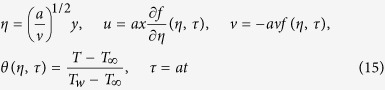


so that (2) and (3) can be written as


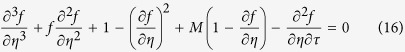



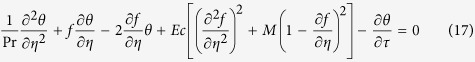


and are subjected to the boundary conditions


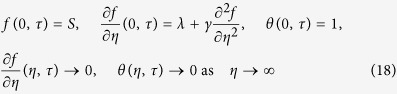


To test the stability of the steady flow solution *f*(*η*) = *f*_0_(*η*) and *θ*(*η*) = *θ*_0_(*η*) satisfying the boundary value problem (1)–(4), we write


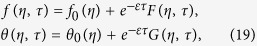


where 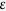 is an unknown eigenvalue, *F*(*η*, *τ*) and *G*(*η*, *τ*) are small relative to *f*_0_(*η*) and *θ*_0_(*η*). Solutions of the eigenvalue problem (16)–(18) give an infinite set of eigenvalues 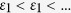; if the smallest eigenvalue is negative, there is an initial growth of disturbances and the flow is unstable but when 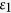 is positive, there is an initial decay and the flow is stable. Introducing (19) into (16) and (17), we get the following linearized problem


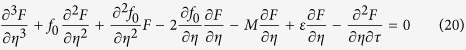



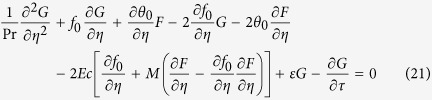


along with the boundary conditions


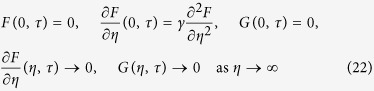


The solutions *f*(*η*) = *f*_0_(*η*) and *θ*(*η*) = *θ*_0_(*η*) of the steady equations (6) and (7) are obtained by setting *τ* = 0. Hence *F*(*η*) = *F*_0_(*η*) and *G*(*η*) = *G*_0_(*η*) in (20) and (21) identify initial growth or decay of the solution (19). In this respect, we have to solve the linear eigenvalue problem


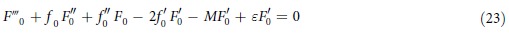



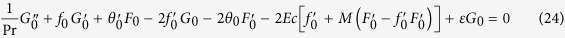


along with the boundary conditions


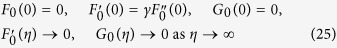


It should be stated that for particular values of Pr and 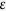, the stability of the corresponding steady flow solutions *f*_0_(*η*) and *θ*_0_(*η*) are determined by the smallest eigenvalue 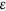. As it has been suggested by Harris *et al.*^24^, the range of possible eigenvalues can be determined by relaxing a boundary condition on *F*_0_(*η*) or *G*_0_(*η*). For the present problem, we relax the condition that 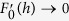 as 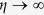 and for a fixed value of *λ* we solve the system (23)–(25) along with the new boundary conditions 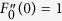.

## Results and Discussion

The system of ordinary differential equations (7) and (8) subjected to the boundary conditions (9) was solved numerically using Runge-Kutta Fehlberg method with shooting technique for some values of the governing parameters. The computation was carried out until we get the velocity and temperature profiles converge and satisfy the far field boundary conditions (9) asymptotically. The numerical calculations were carried out for different values of suction/injection parameter *S*, magnetic parameter *M*, power law stretching/shrinking parameter λ, Prandtl number *Pr*, Eckert number *Ec*, velocity slip parameter γ, and their effects on the flow and heat transfer characteristics are analyzed and discussed.

Table 1 shows the comparison data with those of Aman *et al.*^25^ and Wang^26^ when *M* = 0 and γ = 0 for shrinking case (λ < 0). The stated data clarify the good agreement with the previous data which support the validity of our numerical results.

**Figure 1:**
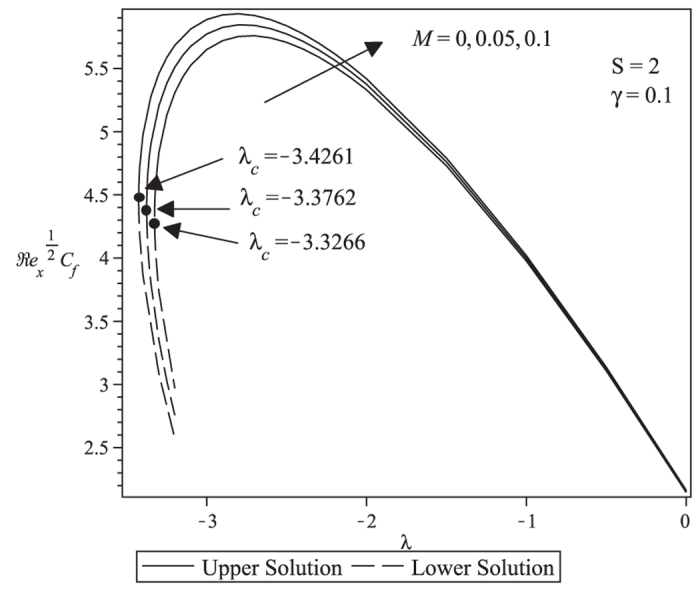
Variation of the skin friction coefficient with *λ* when *Pr* = 1, *Ec* = 0.5, *S* = 2 and *γ* = 0.1 with various value of *M*.

**Figure 2:**
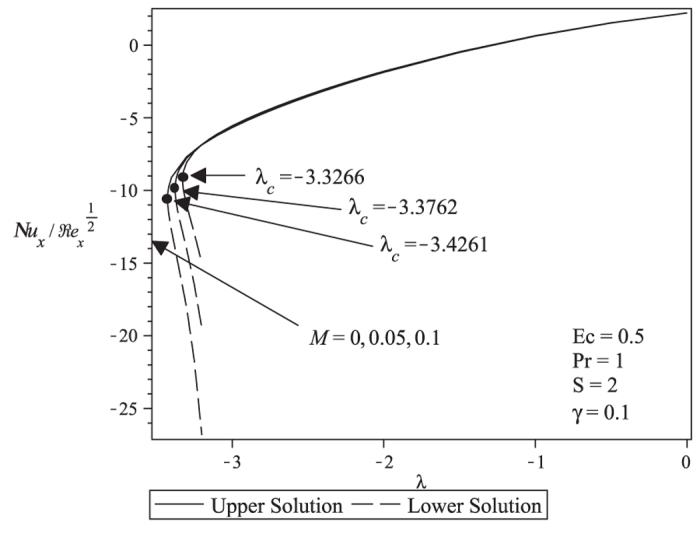
Variation of the local Nusselt number with *λ* when *Pr* = 1, *Ec* = 0.5, *S* = 2 and *γ* = 0.1 with various value of *M*.

Figures 1 and 2 respectively show the variation of the skin friction coefficient C_f_Re_x_^1/2^ and the local Nusselt number Nu_x_/Re_x_^1/2^ (heat transfer rate at the surface) for different values of the magnetic parameter *M*. These figures show that a unique solution exists for equations (7) and (8) with the boundary conditions (9) for the stretching case, dual (upper and lower branch) solutions are found for the shrinking case up to a critical value 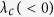, and no solutions exist for 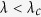. These values of 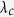 are stated in Figs 1 and 2, which show that increasing the magnetic parameter *M* is to increase the range of *λ* for which the solution exists. The skin friction coefficient and the heat transfer rate at the surface increase as the magnetic parameter *M* increases.

To test the stability of the dual solutions, the stability analysis was performed to find the eigenvalues ε (see Eq. (19)). If the smallest eigenvalue is positive, there is an initial decay and the flow is stable; while if the smallest eigenvalue is negative, there is an initial growth of disturbances and the flow is unstable. The smallest eigenvalues ε for selected values of λ are shown in Table 2. The results indicate that ε is positive for the upper branch solution and negative for the lower branch solution. So, the upper branch solution is stable and physically reliable, while the lower branch is not.

**Figure 3:**
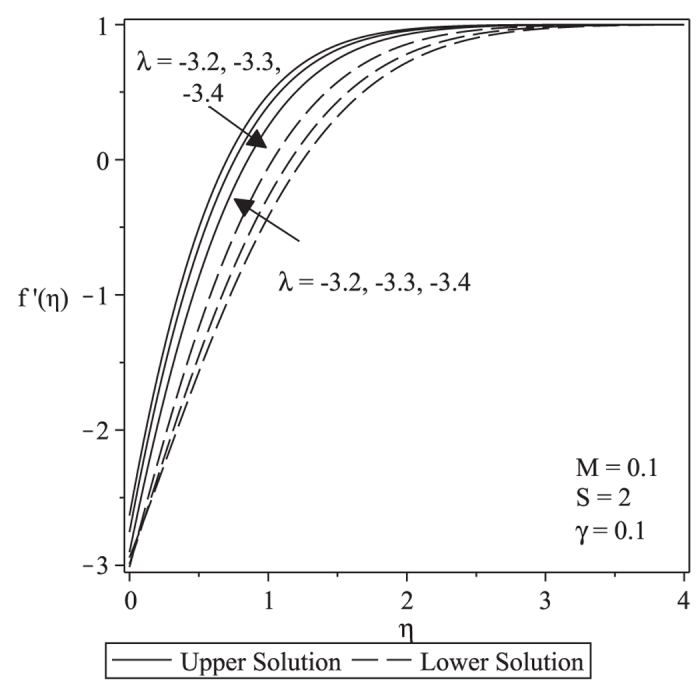
Velocity profiles for different values of λ when M = 0.1, S = 2 and γ =0.1.

**Figure 4:**
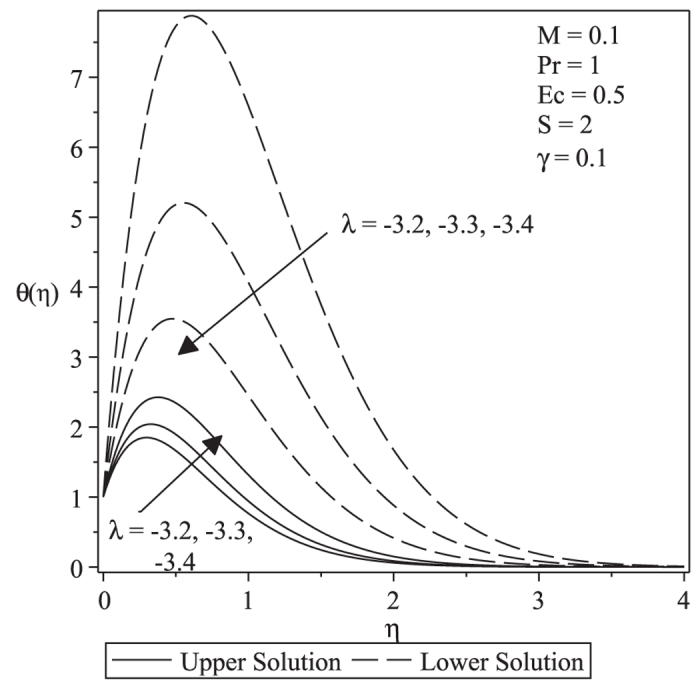
Temperature profiles for different values of *λ* when *M* = 0.1, *Pr* = 1, *Ec* = 0.5, *S* = 2 and *γ* = 0.1.

**Figure 5:**
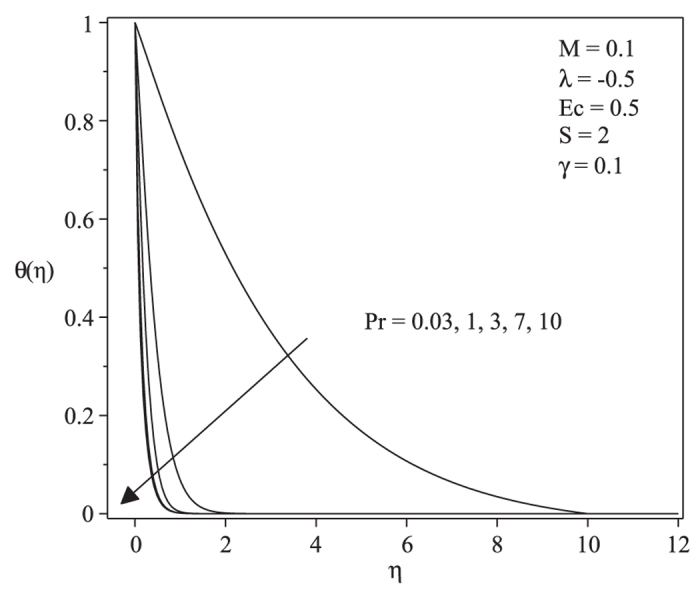
Temperature profiles for different values of *Pr* when *M* = 0.1, *λ* = −0.5, *Ec* = 0.5, *S* = 2 and *γ* = 0.1.

Figures 3 and 4 present the velocity and temperature profiles for different values of λ when *M* = 0.1, *Pr* = 1, *Ec* = 0.5, *S* = 2 and γ = 0.1. From the figures we can see that the boundary layer thickness for the lower solution is thicker compared to upper solution. For a particular value of parameter, there exist two different profiles as presented in Figs 3 and 4, and thus supports the existence of dual solutions in Figs 1 and 2. Figure 5 shows the temperature profile for different values of Pr when the other parameters are fixed. It is seen that the temperature gradient at the surface increases as Pr increases, thus increase the local Nusselt number (heat transfer rate at the surface). This is because increasing Pr will cause the increasing of viscosity, then reduces the thermal conductivity, and thus 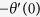 increases (Ishak et al.^27^). All velocity and temperature profiles approach the far field boundary conditions asymptotically which support the validity of the numerical results presented in Figs 1 and 2.

## Conclusion

We have numerically investigated how magnetic parameters influence the effects of viscous dissipation, Joule heating and partial velocity slip. The skin friction coefficient and heat transfer rate at the surface increase as magnetic parameter *M* increases. It was found that there exist dual solutions for the shrinking sheet while only unique solution for the stretching sheet. The stability analysis showed that there is an initial decay for the upper branch solution while there is initial growth of disturbance for the lower branch solution. Thus the upper branch is linearly stable and physically reliable while the lower branch solution is not.

## Additional Information

**How to cite this article**: Yasin, M. H. M. *et al.* MHD Stagnation-Point Flow and Heat Transfer with Effects of Viscous Dissipation, Joule Heating and Partial Velocity Slip. *Sci. Rep.* **5**, 17848; doi: 10.1038/srep17848 (2015).

## Figures and Tables

**Table 1:** Comparison with previously publish data for the values of *f* ″(0), when *M *= 0 and *γ *= 0 (no slip) for shrinking case (*λ* < 0).

*λ*	Present Results		Aman *et al.*^25^		Wang^26^	
	Upper Solution	Lower Solution	Upper Solution	Lower Solution	Upper Solution	Lower Solution
−0.25	1.402241		1.4022		1.40224	
−0.50	1.495670		1.4957		1.49567	
−1.00	1.328817	0	1.3288	0	1.32882	0
−1.10	1.186680	0.049229				
−1.15	1.082231	0.116702	1.0822	0.1167	1.08223	0.11670
−1.18	1.000449	0.178361	1.0004	0.1784		
−1.20	0.932473	0.233650				

**Table 2:** Smallest eigenvalues ε at selected values of λ with various *M* when *S* = 2, *Ec* = 0.5, *Pr* = 1 and γ = 0.1.

M	λ	Upper Solution	Lower Solution
0	−3.2	1.1210	−1.0269
	−3.25	0.8671	−0.8104
	−3.3	0.5061	−0.4864
	−3.32	0.2500	−0.2451
0.05	−3.2	1.2201	−0.9434
	−3.25	0.9682	−0.7265
	−3.3	0.6119	−0.4051
	−3.32	0.3663	−0.1733
0.1	−3.2	1.3169	−0.8570
	−3.25	1.0664	−0.6393
	−3.3	0.7132	−0.3186
	−3.32	0.4730	−0.0913
